# Plin5 alleviates myocardial ischaemia/reperfusion injury by reducing oxidative stress through inhibiting the lipolysis of lipid droplets

**DOI:** 10.1038/srep42574

**Published:** 2017-02-20

**Authors:** Pengfei Zheng, Zhonglin Xie, Yuan Yuan, Wen Sui, Chao Wang, Xing Gao, Yuanlin Zhao, Feng Zhang, Yu Gu, Peizhen Hu, Jing Ye, Xuyang Feng, Lijun Zhang

**Affiliations:** 1 Department of Cardiology, Xijing Hospital, Fourth Military Medical University, Xi’an 710032, Shaanxi Province, China; 2 Department of Cardiology, The Sixteenth Hospital of PLA, Aletai 836500, Xinjiang Province, China; 3 Department of Pathology, Xijing Hospital, Fourth Military Medical University, Xi’an 710032, Shaanxi Province, China; 4 Department of Stomatology Center, Shenzhen Hospital of Southern Medical University, Shenzhen, 518000, Guangdong Province, China; 5 Department of Clinical Diagnosis, Tangdu Hospital, Fourth Military Medical University, Xi’an 710032, Shaanxi Province, China

## Abstract

Myocardial ischaemia-reperfusion (I/R) injury is a complex pathophysiological process. Current research has suggested that energy metabolism disorders, of which the abnormal consumption of fatty acids is closely related, compose the main pathological basis for myocardial I/R injury. Lipid droplets (LD) are critical regulators of lipid metabolism by LD-associated proteins. Among the lipid droplet proteins, the perilipin family members regulate lipolysis and lipogenesis through different mechanisms. Plin5, an important perilipin protein, promotes LD generation and lowers fatty acid oxidation, thus protecting the myocardium from lipotoxicity. This study investigated the protective effects of Plin5 in I/R myocardium. Our results indicated that Plin5 deficiency exacerbated the myocardial infarct area, aggravated left ventricular systolic dysfunction, reduced lipid storage, and elevated free fatty acids. Plin5-deficient myocardium exhibited severely damaged mitochondria, elevated reactive oxygen species (ROS) and malondialdehyde (MDA) levels, and decreased superoxide dismutase (SOD) activity. Furthermore, the decreased phosphorylation of PI3K/Akt in *Plin5*-null cardiomyocytes might contribute to I/R injury aggravation. In conclusion, Plin5, a new regulator of myocardial lipid metabolism, decreases free fatty acid peroxidation by inhibiting the lipolysis of intracellular lipid droplets, thus providing cardioprotection against I/R injury and shedding new light on therapeutic solutions for I/R diseases.

According to the World Health Organization (WHO), ischaemic heart diseases have become the leading causes of human death over the last two decades. At present, coronary reperfusion has been well-established for the treatment of ischaemic myocardium. However, restoration of blood flow to the ischaemic myocardium can lead to the so-called ischaemia/reperfusion (I/R) injury^1^. In animal studies, I/R injury may be responsible for up to 50% of the final infarct size. In 1966, Jennings initially proposed the concept of myocardial ischaemia-reperfusion injury (MI/RI)^2^, and the pathogenesis of I/R injury reflects the confluence of multifactorial aetiologies. However, significant controversies are also present in the field.

The heart is known for its ability to generate energy from fatty acids (FAs) because of its powerful β-oxidation, whereas excess FA oxidation is closely related to increased oxygen consumption that can lead to lipotoxicity. In cardiomyocytes, FA metabolism is strictly regulated to avoid lipotoxicity. To reduce the lipotoxicity generated by FA β-oxidation, excess FAs consumed by cardiomyocytes can be converted into triglyceride (TG) and stored in cytosolic lipid droplets (LDs). Therefore, LDs play a critical role in maintaining lipid metabolism homeostasis.

LDs are considered as metabolically active “organelles” and are involved in lipid storage and transport, intracellular trafficking, signalling, chaperone functions, RNA metabolism, and cytoskeletal organization^3^,^4^. In mammalian cells, LDs have a neutral lipid core including TG or cholesterol esters (CE) and covered by a monolayer of phospholipids. LD surfaces contain anchored proteins that are critical for regulating LD generation, lipolysis and translocation^5^. Among the LD proteins, the perilipin family members are well characterized and regulate lipolysis and lipogenesis through different mechanisms. Perilipin 5 (Plin5) is an important perilipin protein and is abundant in tissues with very active lipid catabolism, such as the heart, liver, skeletal muscle, and brown adipose tissue (BAT)^6^. Consistent with previous studies, we found that Plin5 can inhibit FA oxidation by protecting the LDs against lipolysis in the liver, thus reducing lipotoxicity^7^.

The β-oxidation of long-chain FAs produces the majority of energy required to sustain continuous contractile activity of the heart. Excessive cytosolic FAs can stimulate β-oxidation, which could generate reactive oxygen species (ROS). Medium and high concentrations of ROS induced cell apoptosis can even lead to necrosis through oxidative stress. Detection of malondialdehyde (MDA), the cytotoxic product of lipid peroxidation induced by free radicals, can indicate the degree of lipid peroxidation. As an important antioxidant enzyme, superoxide dismutase (SOD) removes free radicals in the body, reduces the cell damage induced by oxygen free radicals, and repairs damaged cells. Myocardial Plin5 promotes LD generation and lowers the FA oxidation, thus protecting the myocardium from lipotoxic injury^8^. Recent studies of the myocardium indicated that Plin5 is important for heart function by preventing excessive lipolysis, FA oxidation, and oxidative stress^9^. However, several studies have reported that cardiac-specific overexpression of Plin5 (CM-Plin5) provoked cardiac steatosis and increased heart weight, left ventricular hypertrophy, and mild cardiac dysfunction in mice^10^,^11^. The specific mechanism of Plin5 in heart disease is still not clarified. Although excessive Plin5 levels may contribute to cardiomyopathy^12^, Plin5 is also required for normal cardiac metabolism and function. In this study, we investigated whether Plin5 might protect the myocardium from ischaemia-reperfusion injury via lipolysis, FA oxidation, and oxidative stress using established Plin5 deficient mice.

## Results

### Plin5 deficiency exacerbated myocardial infarct size

**Figure 1:**
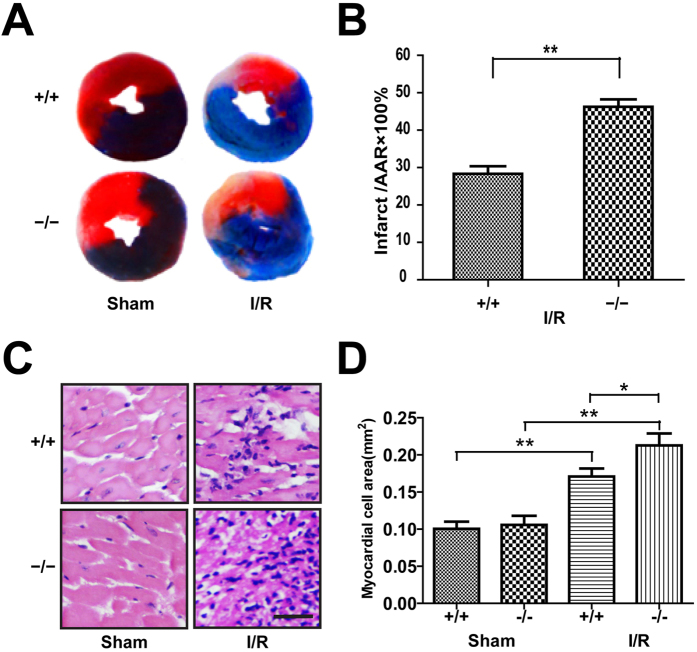
Plin5 deficiency exacerbated the I/R injury of myocardium. (**A**) Mice were sacrificed at the end of reperfusion, and the hearts removed and stained with TTC to measure the myocardial infarct area. (**B**) The infarct size was expressed as a percentage of area at risk (n = 6). (**C**) LV tissues were retrieved at the end of reperfusion, and paraffin sections were prepared and subjected to the H&E staining (n = 4). Representative H&E staining images are shown. Scale bar = 20 μm. (**D**) Myocardial cell size was quantified by using the ipwin32 software. The columns and errors bars represent means ± SEM. *P < 0.05; **P < 0.01.

After 40 min of ischaemia and 24 h of reperfusion, the mouse hearts were dissected and dyed with triphenyl tetrazolium chloride (TTC) and Evans blue. The myocardial infarct size was quantified by calculating the TTC ratio of the stain negative area (infarcted tissues) to the TTC stained area (ischaemic but viable tissue) (Fig. 1A). The results indicated that the myocardial infarct size of the *Plin5*-null mice was significantly increased compared to that of the wide-type mice with I/R surgery (46.29 ± 2.397% vs. 28.33 ± 2.516%; *P* < 0.01). However, no infarct area was found in the wild-type and *Plin5*-null mice that underwent sham surgery (Fig. 1B).

The myocardial fibres of the wild-type and *Plin5*-null mice were uniformly textured in the sham surgery groups as observed using microscopy. However, I/R injury did damage the myocardial tissues, by increasing swelling, myofibre breakage, inflammatory cell infiltration, bleeding into the myocardial interstitials, and accumulation of necrotic myocardial nuclear clusters (Fig. 1C). Moreover, the myocardial damage in the *Plin5*-null myocardium was more severe than that observed in wild-type mice after I/R surgery. We quantified the cross-sectional size of the myocardial cells using the ipwin32 software and found that the swelling in the *Plin5*-null myocardium was more obvious than that in wild-type mice after I/R surgery (0.2241 ± 0.0620 vs. 0.1768 ± 0.0438; *P* < 0.05) (Fig. 1D).

### Plin5 deficiency aggravated I/R heart dysfunction

**Figure 2:**
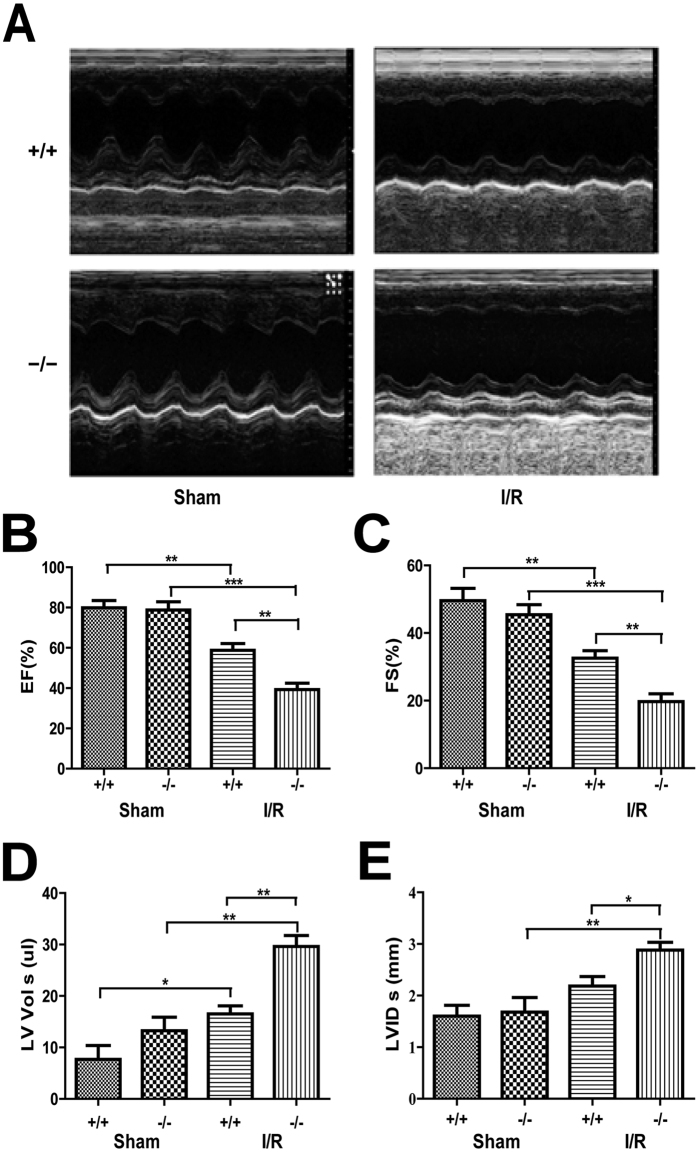
Plin5 deficiency aggravates heart dysfunction following I/R injury. (**A**) Echocardiography was performed at the end of reperfusion, and representative M-mode echocardiograms were recorded in all groups. Mice without LAD occlusion served as basal controls (Sham group). (n = 6). (**B**–**E**) Cardiac function was examined by echocardiography after I/R surgery. The EF (**B**) and FS (**C**) values of *Plin5*-null mice decreased more significantly than those in wild-type mice after I/R surgery, whereas LV Vol;s (**D**) and LVID;s (**E**) also increased significantly in *Plin5*-null mice after I/R surgery. (n = 6). The columns and errors bars represent means ± SEM. *P < 0.05; **P < 0.01; ***P < 0.001. EF, ejection fraction; FS, fractional shortening; LV Vol;s, left ventricular contraction volume; LVID;s, left ventricular internal diameter at end-systole.

Left ventricular function was evaluated using echocardiography after 40 min of ischaemia and 24 hour of reperfusion. After sham surgery, no significant difference in baseline echocardiography was observed between the wild type and *Plin5*-null mice (Fig. 2A). However, the I/R injury caused significant changes in systolic function in both wild-type and *Plin5*-null mice, including ejection fraction (EF), fractional shortening (FS), left ventricular contraction volume (LV Vol;s) and left ventricular internal diameter at end-systole (LVID;s). The EF and FS values were decreased in I/R mice (Fig. 2B,C). However, the contraction functions of *Plin5*-null mice decreased more significantly than in wild-type mice after I/R surgery (EF: 39.31 ± 4.396% vs. 58.76 ± 4.816%, *P* < 0.01; FS: 19.71 ± 3.267% vs. 32.56 ± 3.134%, *P* < 0.01). At the same time, I/R injury caused a significant diastolic insufficiency with increasing LV Vol;s and LVID;s in *Plin5*-null mice compared with those in wild-type mice after I/R surgery (LV Vol;s: 29.62 ± 3.005 vs. 16.52 ± 2.199, *P* < 0.01; LVID;s: 2.880 ± 0.2166 vs. 2.185 ± 0.0.2576, *P* < 0.05) (Fig. 2D,E). These data indicated that Plin5 deficiency can aggravate heart dysfunction following I/R injury.

### Plin5 deficiency damaged mitochondria in the I/R myocardium

**Figure 3:**
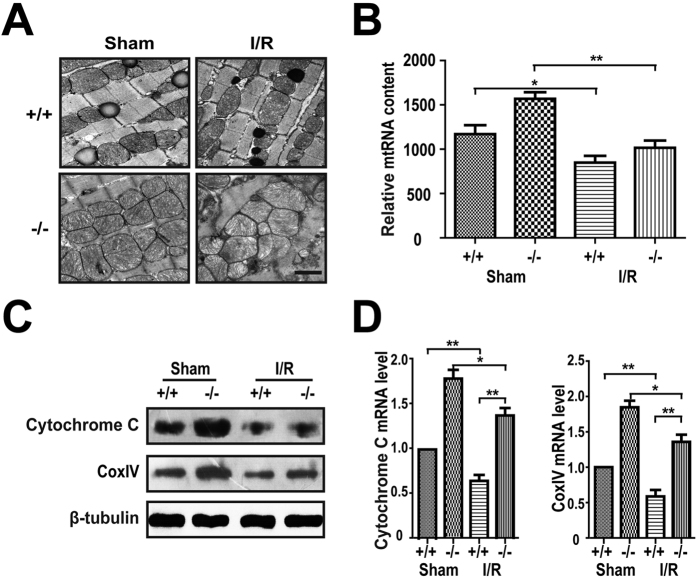
Plin5 deficiency enhanced proliferation of mitochondria in myocardium with I/R injury. (**A**) Electron microscopy images of mouse myocardium with or without I/R injury (n = 4). Mitochondrial sizes were analysed using Image pro plus 6.0 software in four electron microscopic images of the myocardium from wild-type and *Plin5*-null mice. Scale bar = 1 μm. (**B**) Relative mitochondrial DNA (mtDNA) contents (normalised to the single-copy nuclear gene Ndufv1) in the myocardium of wild-type and *Plin5*-null mice (n = 4). (**C**) Immunoblotting analysis of the levels of mitochondria markers in the myocardium of mice (n = 4). (**D**) Quantitative PCR analysis of the mRNA levels of mitochondria markers in the myocardium with or without I/R injury (n = 4). The columns and errors bars represent the means ± SEM. *P < 0.05, **P < 0.01.

FAs are oxidized in mitochondria, and the mitochondrial function and structure are critical to the utilization of FAs in the myocardium. To further determine the roles of Plin5 in myocardial I/R injury, we observed the ultrastructural changes in myocardial tissues following I/R injury using electron microscopy. For the mice that underwent sham surgery, the myofibrillar bundles of the Z and M lines of the myocardium were relatively clear. However, the number of mitochondria increased significantly in the *Plin5*-null myocardium from the mice that sham surgery, which indicated that Plin5 deficiency could promote mitochondrial proliferation. After I/R surgery, both the wild-type and *Plin5*-null myocardial mitochondria became deformed and showed increased oedema, reduced matrix density, and vague mitochondrial cristae. However, the mitochondrial damage was severe in *Plin5*-null mice than in wild-type mice (Fig. 3A). We found that the mtDNA content also increased significantly in *Plin5*-null myocardium without ischaemia reperfusion (Fig. 3B). Moreover, the immunoblotting results indicated that the mitochondrial markers cytochrome C and cytochrome C oxidase subunit IV (COX IV) increased significantly in *Plin5*-null myocardium compared with wild-type mice with and without ischaemia reperfusion (Fig. 3C), which was consistent with the results of quantitative polymerase chain reaction (PCR) (Fig. 3D). These results indicated that Plin5 deficiency could promote the proliferation of myocardial mitochondria and the deterioration of mitochondria after I/R injury.

### Plin5 deficiency reduced lipid storage and increased ROS damage following I/R injury

**Figure 4:**
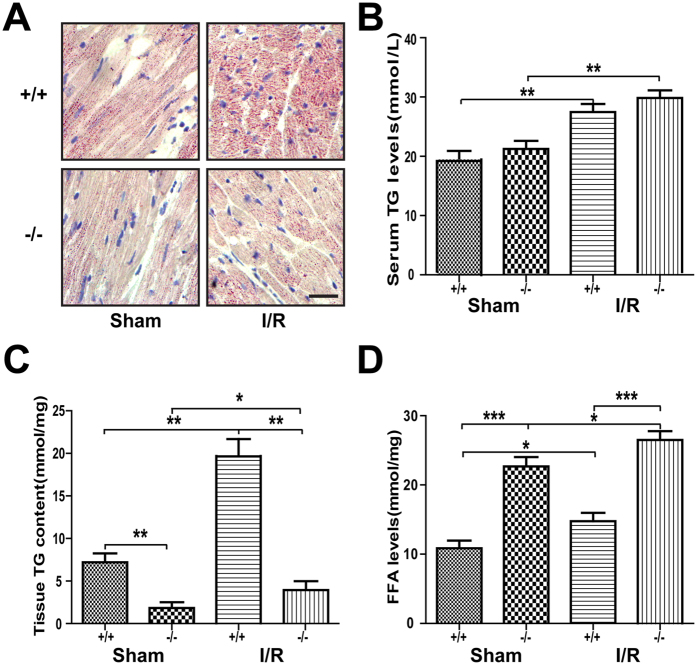
Plin5 deficiency reduced lipid storage and elevated the cytosolic fatty acid levels in myocardium with I/R injury. (**A**) Oil Red O staining showed that Plin5 deficiency led to reduced cardiac lipid accumulation in mice with either sham or I/R surgery (n = 4). Scale bar = 20 μm. (**B**–**D**) Quantitative analysis of serum TG levels (**B**), myocardial TG contents (**C**), and FFA contents (**D**) (n = 4). Serum TG levels were obviously increased after I/R injury, but no difference was observed between wild-type and *Plin5*-null mice. (**C**) The amounts of TG storage decreased slightly, whereas the levels of intracellular FFA (**D**) increased in *Plin5*-deficient myocardium. The columns and errors bars represent the means ± SEM. *P < 0.05, **P < 0.01, ***P < 0.001. FFA, free fatty acid.

In this study, we measured the blood glucose levels of each group and found that the blood glucose was not changed in the *Plin5*-null and wild-type mice, although the I/R surgery increased the blood glucose compared with that of the sham surgery group (data not shown). Moreover, we found that the blood TG levels were obviously increased after I/R, whereas they were not different between the wild-type and *Plin5*-null mice (Fig. 4B). However, Oil Red O staining showed that the myocardial lipid content decreased in *Plin5*-null mice compared with that in wild-type mice following either sham or I/R surgery (Fig. 4A), which indicated decreased lipid accumulation in *Plin5*-null hearts. Similar to Oil Red O staining, the LD number and size in *Plin5*-null myocardium decreased when compared with that wild-type mice as observed using electron microscope (Fig. 3A). According to the results of TG quantification in the myocardial tissues, we found that the myocardial TG contents were increased after I/R (*P* < 0.05) in both *Plin5*-null and wild-type mice, whereas TG contents in the *Plin5*-null myocardium were significantly lower than those in wild-type mice (Fig. 4C). Interestingly, we found that the free fatty acid (FFA) contents in the myocardium were higher in *Plin5*-null mice than in wild-type mice after either sham or I/R surgery (Fig. 4D). Summarily, Plin5 deficiency reduced lipid storage in myocardium and elevated the cytosolic FFA levels.

**Figure 5:**
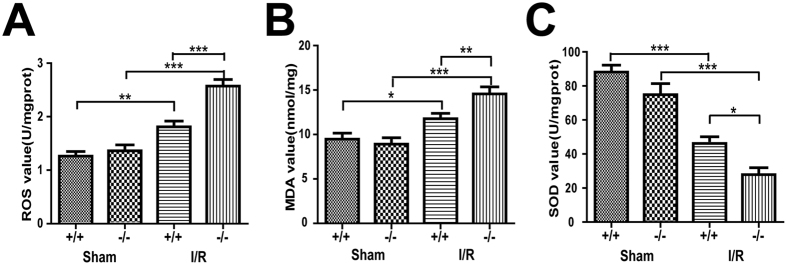
Plin5 deficiency increased the ROS levels in I/R myocardium. Cardiac levels of ROS (**A**), MDA (**B**) and SOD (**C**) (n = 4). The ROS and MDA levels of the I/R myocardium increased more so than in wild-type mice, whereas the SOD activity decreased in *Plin5*-null mice. The columns and errors bars represent the means ± SEM. *P < 0.05, **P < 0.01, ***P < 0.001. ROS, reactive oxygen species; MDA, malondialdehyde; SOD, superoxide dismutase.

Our results indicated that the MDA levels and ROS activities were similar between the myocardium of the *Plin5*-null and wild-type mice with sham surgery, whereas the MDA levels and ROS activities of the I/R myocardium were higher in the *Plin5*-null mice than in the wild-type mice (Fig. 5A, B). However, as the critical enzymatic scavenger that limits damage from ROS, SOD activity in the I/R myocardium decreased in *Plin5*-null mice compared with that in wild-type mice (Fig. 5C). These data suggested that Plin5 deficiency could increase ROS levels in the I/R myocardium.

### Plin5 deficiency decreased the phosphorylation of AKT and PI3K in the I/R myocardium

**Figure 6:**
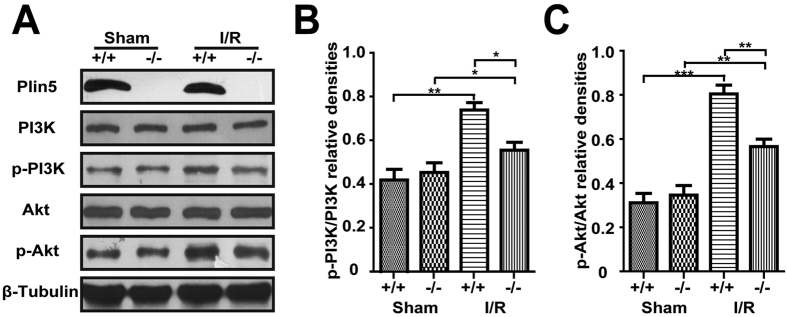
Plin5 played a protective role following ischaemia reperfusion in the myocardium by activating the PI3K/Akt signalling pathway. The levels of total PI3K, Akt and their phosphorylated forms (p-PI3K, p-Akt) were assessed using Western blot (n = 4). (**A**) Representative results for western blot analysis. (**B**,**C**) Semi-quantitative analysis of the relative expression levels of PI3K and p-PI3K, Akt and p-Akt in each group of mice were normalized against those of β-tubulin and are presented as a ratio between p-PI3K/PI3K (**B**) and p-Akt/Akt (**C**). The columns and errors bars represent the means ± SEM. *P < 0.05, **P < 0.01, ***P < 0.001.

We also examined the expression and phosphorylation of class I phosphoinositide 3-kinase (PI3K) and serine/threonine protein kinase (Akt). I/R injury increased the expression of phosphorylated PI3K and Akt in the heart tissue of both *Plin5*-null and wild-type mice, whereas Plin5 deficiency reduced the phosphorylation of PI3K and Akt in the myocardium (*P* < 0.05, Fig. 6A). These results indicated that Plin5 may activate the PI3K/Akt signalling pathway. Moreover, similar trends in the semi-quantitative analysis of the relative expression levels of these genes were also observed (Fig. 6B,C). Summarily, these data indicated that decreased phosphorylation of PI3K/Akt in *Plin5*-null cardiomyocytes might contribute to the aggravation of I/R injury.

## Discussion

Multiple factors and mechanisms are involved in the pathogenesis of myocardial I/R injury^13^. The oxidation of FAs consumes significant amounts of oxygen, which can exacerbate I/R injury, and previous studies have indicated that excessive FA oxidation elevates oxidative damage^14^. Under physiological conditions, the energy from FAs that drives for cardiac activity is more than 60%^15^. During ischaemia and early reperfusion, the oxygen supply is insufficient, and heart energy metabolism shifts from mitochondrial aerobic oxidation to glycolysis to maintain myocardial cell survival^14^. Enhanced glycolysis increases the generation of lactate and decreases the intracellular pH^15^. Meanwhile, the enzymes involved in the generation of ROS are unregulated and activated, and the electron transfer chain is also turned on. This generates ROS after the myocardial blood supply is restored^16^,^17^. Elevated ROS leads to peroxidation of membrane lipids, denaturation of proteins and deactivation of enzymes, which together with myofilament loss ultimately leads to myocardial cell damage and death. Therefore, the decline in FA oxidation during the myocardial I/R period inhibits the production of ROS and reduces the myocardial injury area.

LDs are regarded as an important “organelles” for regulating lipid metabolism and can store most excess FAs as TG. However, the regulation of LD metabolism in these tissues remain poorly understood. The proteins anchored on the LD surfaces are known as LD-associated proteins, which are involved in the formation, maturation, secretion, and trafficking of LDs and participate in regulating lipolysis and lipogenesis^18^. Plin5, which is also known as MLDP (Myocardial lipid droplet protein), OXPAT (Oxidative tissue enriched PAT protein), and LSDP5 (Lipid storage droplet protein 5) according to the initial identifications and characterizations^6^,^8^,^19^,^20^, is an important member of the perilipin family. Plin5 is enriched in the heart and can inhibit the lipolysis of LD and then reduce lipid oxidation^21^.

Here, we studied the roles of Plin5 in myocardial I/R injury, and the results indicated that Plin5 deficiency aggravated myocardial I/R injury. We found that Plin5 deficiency did not affect blood TG and glucose levels, although I/R surgery slightly increased these levels. However, Plin5 deficiency affected lipid metabolism in I/R myocardium by reducing lipid storage and elevating the cytosolic FA contents in the myocardium. We found that in the myocardium, the TG content was significantly lower, and the FFA content was higher in *Plin5*-null mice than in wild-type mice. These results indicated that Plin5 deficiency could reduce the lipid storage in myocardium and elevate the cytosolic FFA levels. A recent study showed that when LD lipolysis was increased in *Plin5*-null myocardium, the generated FAs would be used as fuel for β-oxidation^9^. In our previous study, Plin5 deficiency enhanced lipolysis and free FA oxidation in hepatocytes^7^. Taken together, we speculated that Plin5 deficiency could increase the lipolysis of LD in the myocardial I/R injury.

Moreover, we found that the ROS levels increased in the I/R myocardium of *Plin5*-null mice, which indicated that Plin5 deficiency could aggravate the ROS-mediated damage. Due to the decreased oxygen supply to I/R myocardium, the FA oxidation was substituted for glycolysis, and the excessive FAs were converted into TG to reduce lipotoxicity. The elevated FFAs stimulated the FA oxidation, which exacerbated oxygen consumption and generated more ROS. As a product of lipid peroxidation, MDA is induced by oxygen free radicals, and its level may reflect the degree of cell damage, whereas SOD can scavenge superoxide anions and protect cells from damage. After reperfusion, the level of MDA in the *Plin5*-null myocardial tissue was significantly higher than that in the wild-type myocardial tissue, which paralleled the ROS levels. All of these results suggested that Plin5 inhibits ROS production, reduces myocardial infarct size, and protects the I/R myocardium.

Our previous study found that both the protein and mRNA levels of peroxisome proliferator-activated receptor (PPAR) α increased significantly in *Plin5*-deficient livers fed either a normal diet (ND) or a high-fat diet (HFD)^7^. The PPAR family is regulated by intracellular FAs and their derivatives^22^. PPARs may bind to peroxisome proliferator response elements (PPREs) together with the retinoid-X-receptor (RXR) and therefore may increase the FA oxidation capacity. It has been reported that fenofibrate, a PPARα agonist, significantly suppressed ERK1/2 and Akt activation induced by high glucose^23^. Activated Akt is a downstream effector of PI3K, which can inhibit apoptosis by regulating multiple targets such as mitochondrial permeability transition pore (MPTP), ATPase, tumour necrosis factor alpha (TNF-α), endothelial nitric oxide synthase (eNOS), the Bcl-2 family proteins, and NF-κB^24^. The PI3K/Akt pathway is an important antiapoptosis/proliferation signalling pathway that plays a key role in normal cellular functioning, including proliferation, adhesion, migration, invasion, energy metabolism, and protein synthesis^25^. In addition, many studies have shown that the activation of the PI3K/Akt signalling pathway can suppress apoptosis and excessive autophagy, thus protecting the heart against myocardial I/R injury and regulating mitochondrial function^26^,^27^,^28^,^29^,^30^. In this study, we found that the PIK3/Akt signalling pathway was suppressed in *Plin5*-null mice compared with wild-type mice, although the specific mechanism behind this process is not clear. Therefore, we speculate that Plin5 may activate the PI3K/Akt signalling pathway by suppressing PPARα, which requires further study. In conclusion, cardioprotection of Plin5 was realized by improvement in mitochondrial energy metabolism, prevention of oxidative stress, amelioration of intracellular lipolysis and regulation of the PI3K-related signalling pathway.

In summary, we have demonstrated that Plin5, a new regulator of myocardial lipid metabolism, provides obvious cardioprotection against I/R injury and sheds new lights on auxiliary therapeutic solutions for I/R diseases.

## Materials and Methods

### Generation, Genotyping and Maintenance of Plin5-null mice

All animal experiments were conducted in accordance with the Guidelines for the Care and Use of Laboratory Animals of the Fourth Military Medical University (Shaanxi, China). All study protocols were approved by the Committee of the Fourth Military Medical University (Shaanxi, China). *Plin5*-null (*Plin5*^−/−^) mice were generated using the standard gene disruption procedure as previously described^7^ and backcrossed with C57BL/6 mice for 7 generations. Wild-type and *Plin5*-null mice were obtained by mating heterozygous parents. The animals were maintained under standard conditions at 22–26 °C with a 12-h light/12-h dark cycle and with ad libitum access to water and chow diet. Mouse genotypes were determined using routine PCRs, and the genomic DNA was isolated from mouse tails.

To observe whether Plin5 protects against cardiac I/R injury in mice via lipolysis, FA oxidation, and oxidative stress, mice were randomly divided into the following groups with n = 34 each: (1) *Plin5*^+/+^ + Sham; (2) *Plin5*^+/+^ + I/R; (3) *Plin5*^−/−^ + Sham; and (4) *Plin5*^−/−^ + I/R. For the generation of I/R injury, 12-week-old male mice were anesthetized with 2% isoflurane. Myocardial ischaemia was produced by temporarily exteriorizing the heart via a left thoracic incision and placing a 6–0 silk suture slipknot around the left anterior descending (LAD) coronary artery. After 40 min of ischaemia, the slipknot was released, and the myocardium was reperfused for 24 h. Sham operation control mice underwent the same surgical procedures, except that the suture placed under the LAD was not tied^31^.

### Echocardiography

First, the mice (n = 6) were anesthetized with isoflurane 24 h after MI/R operation. Then, two-dimensional and M-mode echocardiographic measurement was performed with a VEVO 770 high-resolution *in vivo* imaging system (Visual Sonics, Toronto, Canada). All measurements represent the mean of 5 consecutive cardiac cycles. Left ventricular ejection fraction (EF), left ventricular fractional shortening (FS), left ventricular contraction volume (LV Vol;s), and left ventricular internal diameter at the end-systole (LVID;s) were calculated using computer algorithms. All of these measurements were performed in a blinded manner.

### Quantitative Determination of Myocardial Infarct Size

At the end of the 24 h reperfusion and after cardiac function assessment, the LAD ligature was retied, and 2% Evans blue dye was injected into the left ventricular cavity (n = 6). The heart was quickly excised, frozen at −20 °C, and sectioned into 1 mm planes perpendicular to the long axis of the heart. The slices were individually incubated in 24-well culture plates in 1% TTC in phosphate buffer (pH 7.4) at 37 °C for 10 min and photographed. The Evans blue-stained area (area not at risk, ANAR), the TTC stained area (red colour, ischaemic but viable tissue), and the TTC stained negative area (white colour, infarcted tissue) were digitally measured using an IP Lab Image Analysis Software (Version 3.6; Scanalytics). The myocardial infarct size was expressed as a percentage of the infarct area over the area-at-risk (AAR)^31^,^32^.

### Myocardial MDA and ROS levels and SOD activity measurement

After the experiment, the hearts (n = 4) were rapidly removed, and part of the ischaemic myocardium was dissected and flushed with 4 °C saline. The tissues were homogenized with phosphate buffered saline (PBS) (pH 7.4). After centrifugation at 12,000 × g for 30 min, the MDA and ROS levels and SOD activity were measured using commercial kits (Nanjing Jiancheng Bioengineering Institute, China) following the manufacturer’s instructions.

### Western Blotting

For immunoblotting, frozen AAR samples of the mouse left ventricular anterior wall (n = 4) were homogenized in lysis buffer (25 mM Tris-HCl, pH8.0, 150 mM NaCl, 2 mM EDTA, 1% sodium deoxycholate, 0.5% SDS, 1% Triton X-100, and proteinase inhibitor cocktail), and the lysates were centrifuged for 30 min at 12,000 × g to remove cell debris. Protein concentration was determined with the Bio-Rad protein assay kit (Bio-Rad, USA). Aliquots of the extracts were subjected to SDS-PAGE and transferred to PVDF membranes. Primary antibodies against phospho-PI3K (p-PI3K), PI3K, phospho-Akt (p-Akt), Akt, COXIV, and horseradish-peroxidase (HRP-) linked anti-rabbit antibody purchased from Cell Signaling Technology were used for Western blot analysis. Cytochrome C antibody was purchased from BD Pharmingen. The affinity-purified anti-Plin5 monoclonal antibody was generated by our lab^7^. Proteins were probed with the antibody and detected by enhanced chemiluminescence (ECL) (Amersham Biosciences).

### HE staining and myocardial morphology analysis

After reperfusion, the hearts (n = 4) were placed in 10% buffered formalin. Paraffin-embedded left ventricular anterior wall tissues were sectioned into 5 μm thick serial slices and stained with HE. The slides were observed and photographed under a microscope.

### Lipid Analysis

Cryopreserved mouse left ventricular anterior wall tissue (100 mg) was homogenized in ice-cold PBS (1:20, w/v). The total lipids were extracted as previously described^33^. Briefly, 2 ml heart homogenate was added to 8 ml methanol/chloroform (2:1, v/v). After vigorously mixing several times, the tubes were centrifuged at 3,000 × g for 5 min. The lipid-containing lower phase was removed, placed in vials and dried under nitrogen steam. The lipids were resolved in 200 μl 2% Triton X-100 and assayed using a triglyceride kit (Wako Pure Chem, Japan). TG contents were normalized to the heart tissue weight (mg). FFA levels of the heart tissue samples were measured using commercial assay kits (Applygen Technologies, China) following the manufacturer’s instructions (n = 4).

For Oil Red O staining, the mice were sacrificed (n = 4), and the left ventricular anterior wall tissues were immediately removed, cut into 10 μm sections and fixed in 10% formalin for 10 min. The sections were washed in 60% (v/v) isopropanol for 2 min, stained with 0.5% (w/v) Oil Red O solution in 60% isopropanol for 30 min, washed briefly with 60% isopropanol and subsequently with PBS and then counter-stained with haematoxylin prior to microscopy.

### Myocardial cell ultrastructure evaluation

The I/R myocardial tissues of the left ventricular anterior wall were cut into 1 mm^3^ small pieces (n = 4). The dissected tissues were prefixed with 2.5% cold glutaraldehyde overnight at 4 °C. After being washed with PBS, the tissues were fixed with 1% osmiumtetroxide for 1 h. After dehydration in a series of acetone gradients, the specimens were embedded in Epon 812 resin, and ultra-thin sections (70 nm) were cut and placed onto slides. The sections were stained with uranyl acetate and lead citrate for JEM-1011 transmission electron microscopy.

### Determination of mitochondrial DNA copy number

Total left ventricular anterior wall tissue DNA was extracted using the Universal Genomic DNA Extraction Kit (TaKaRa, Japan). The mtDNA copy number was determined using real-time PCR as previously reported^34^. The relative mtDNA content was determined using the ΔCT method. Primers for the mtDNA-encoded CO1 gene were (forward) 5′-TGC TAG CCG CAG GCA TTA C-3′ and (reverse) 5′-GGG TGC CCA AAG AAT CAG AAC-3′, and primers for the single-copy nuclear gene Ndufv1 were (forward) 5′-CTT CCC CAC TGG CCT CAA G-3′ and (reverse) 5′-CCA AAA CCC AGT GAT CCA GC- 3′. The results are presented as the ratio of mtDNA relative to nuclear DNA (n = 4).

### Quantitative Real-time PCR

Total RNA was isolated from left ventricular anterior wall tissues with TRIzol (Invitrogen, USA) according to the manufacturer’s instructions. RNA concentration and quality was determined using a Nanodrop ND-2000 spectrophotometer (NanoDrop Technologies, USA). Total RNA was reverse transcribed into cDNA using PrimeScript RT Master Mix kit (TaKaRa, Japan), and real-time PCR was performed using the SYBR Premix Ex Taq II (TaKaRa, Japan) with an ABI StepOne Plus Real-time PCR system (Applied Biosystems, USA). Each analysis was performed in three to six replicates. The primers for real-time PCR for the Cyc gene were (forward) 5′–CCA AAT CTC CAC GGT CTG TTC-3′ and (reverse) 5′ –ATC AGG GTA TCC TCT CCC CAG-3′ and primers for the CoxIV gene were (forward) 5′–CGG CGT GAC TAC CCC TTG-3′ and (reverse) 5′–TGA GGG ATG GGG CCA TAC A-3′. Relative gene expression was normalized to the reference gene β-actin or 18 S rRNA using the ΔΔCT method (n = 4).

### Statistical Analysis

All data are presented as the mean ± SEM. The statistical significance of the differences between groups was assessed by Student’s *t*-test using SPSS 13.0 software (Chicago, IL, USA). The criterion for significance was set at *P  * <0.05.

## Additional Information

**How to cite this article**: Zheng, P. *et al*. Plin5 alleviates myocardial ischaemia/reperfusion injury by reducing oxidative stress through inhibiting the lipolysis of lipid droplets. *Sci. Rep.* **7**, 42574; doi: 10.1038/srep42574 (2017).

**Publisher's note:** Springer Nature remains neutral with regard to jurisdictional claims in published maps and institutional affiliations.
